# Assessing the impact of an intensive dietitian-led telehealth intervention focusing on nutritional adequacy, symptom control and optional supplemental jejunal feeding, on quality of life in patients with pancreatic cancer: a randomised controlled trial protocol

**DOI:** 10.1186/s12937-025-01113-9

**Published:** 2025-04-07

**Authors:** Emma McShane, Kate Furness, Lauren Hanna, Kate Connell, Terrence Haines, Catherine E. Huggins, John Zalcberg, Sharon Carey, Charles H.C. Pilgrim, Joanne Lundy, Andrew Metz, David Kissane, Michael Franco, John Coutsouvelis, Diederick W. De Boo, J Simon Bell, Mahesh Iddawela, Theresa Dodson, Ignatius Pereira, Nina Imad, Jill Kirkpatrick, Cherie Dear, Daniel Croagh

**Affiliations:** 1 Nutrition & Dietetics Department, Alfred Health, Melbourne, VIC Australia; 2 Department of Sport, Exercise and Nutrition Sciences, School of Allied Health, Human Services and Sport, La Trobe University, Bundoora, VIC Australia; 3 Department of Nutrition, Dietetics and Food, Faculty of Medicine Nursing and Health Sciences, Monash University, Notting Hill, VIC Australia; 4 School of Primary and Allied Health Care, Faculty of Medicine, Nursing and Health Sciences, Monash University, Frankston, VIC Australia; 5 Institute for Health Transformation, Global Centre for Preventive Health and Nutrition, School of Health and Social Development, Deakin University, Geelong, VIC Australia; 6 Department of Medical Oncology, Alfred Health, Melbourne, VIC Australia; 7 School of Public Health and Preventive Medicine, Faculty of Medicine, Nursing and Health Sciences, Monash University, Clayton, VIC Australia; 8 Nutrition and Dietetics Department, Royal Prince Alfred Hospital, Camperdown, NSW Australia; 9 Hepaticopancreaticobiliary Surgery Unit, Alfred Health, Melbourne, VIC Australia; 10 Department of Medical Oncology, Peninsula Health, Frankston, VIC Australia; 11 Department of Medicine, Peninsula Clinical School, Monash University, Frankston, VIC Australia; 12 Department of Gastroenterology, Royal Melbourne Hospital, Parkville, VIC Australia; 13 School of Medicine, University of Notre Dame Australia, Sydney, NSW Australia; 14 Department of Psychiatry, Monash University, Clayton, VIC Australia; 15 St Vincent’s Health, Fitzroy, VIC Australia; 16 Centre for Medication Use and Safety, Faculty of Pharmacy and Pharmaceutical Sciences, Monash University, Parkville, VIC Australia; 17 Pharmacy Department, Alfred Health, Melbourne, VIC Australia; 18 Department of Radiology, Monash Medical Centre, Monash Health, Clayton, VIC Australia; 19 Department of Medical Imaging and Radiation Sciences, School of Clinical Sciences, Faculty of Medicine, Nursing and Health Sciences, Monash University, Clayton, VIC Australia; 20 Latrobe Regional Health, Traralgon, VIC Australia; 21 Upper Gastrointestinal and Hepatobiliary Surgery, Monash Medical Centre, Monash Health, Clayton, VIC Australia; 22 Department of Nursing and Allied Health, School of Health Sciences, Swinburne University of Technology, Hawthorn, VIC Australia; 23 Consumer Representatives, Melbourne, VIC Australia; 24 Department of Surgery, School of Clinical Sciences, Faculty of Medicine, Nursing and Health Sciences, Monash University, Clayton, VIC Australia; 25 School of Translational Medicine, Faculty of Medicine, Nursing and Health Sciences, Monash University, Clayton, VIC Australia; 26 Faculty of Medicine, Nursing and Health Sciences, Monash University, Clayton, VIC Australia; 27 Faculty of Information Technology, Monash University, Clayton, VIC Australia; 28 The University of Melbourne, Parkville, VIC Australia; 29 Jreissati Pancreatic Centre, Epworth HealthCare, Richmond, VIC Australia

**Keywords:** Malnutrition, Pancreatic cancer, Nutrition impact symptoms, Randomised controlled trial, Pancreatic exocrine insufficiency, Dietitian, Nutrition, Tube feeding

## Abstract

**Background:**

Pancreatic cancer is the third leading cause of cancer-related death in Australia, with a persistently poor 5-year survival rate of around 13%. Symptoms arising from the disease and chemotherapy such as epigastric pain, anorexia, bloating and fat-malabsorptive diarrhoea cause poor oral intake and weight loss, and reduce an individual’s quality of life and ability to tolerate anti-cancer treatment. The primary aim of this study is to determine if an early, intensive telehealth nutrition intervention can improve quality of life compared to usual care for people undergoing treatment for pancreatic cancer.

**Methods:**

This multicentre randomised controlled trial will recruit adults newly diagnosed with borderline resectable, locally advanced or metastatic pancreatic cancer from multiple health services across Victoria (metropolitan and regional). The control group will receive usual nutrition care, which is site-dependent. The intervention group will receive weekly telehealth dietetic consultations for six months, targeting nutritional adequacy through dietary education and counselling, oral nutrition supplement drinks and dietetics-led symptom management advocacy, including appropriate dosing of pancreatic enzymes. Escalation to supplemental jejunal tube feeding may occur if clinically required in the intervention arm. The primary outcome is quality of life (EORTC-QLQ C30 summary score); secondary outcomes include survival, chemotherapy dosing changes, and nutrition status markers including body composition. Outcomes will be measured at baseline, and three- and six-months.

**Discussion:**

The findings of this study will provide evidence of the impact that intensive nutrition therapy, including counselling, provision of oral nutrition supplement drinks and the option for jejunal feeding, has on quality of life and health outcomes in pancreatic cancer. The consistent dietetic approach with the use of telehealth consultations to reduce malnutrition and aid symptom management challenges the current model of care.

**Trial registration:**

31st January 2024, Australian and New Zealand Clinical Trial Registry (Trial ID/No. ACTRN12624000084583).

## Background

Pancreatic cancer is the third leading cause of cancer-related death in Australia, and predicted to be the second leading by 2030, with a persistently poor 5-year survival rate of around 13% [1]. The presence of debilitating symptoms related to pancreatic cancer such as epigastric pain, bloating, loss of appetite, and fat-malabsorptive diarrhoea causes poor oral intake and weight loss, with 80% of patients reporting weight loss prior to diagnosis [2, 3]. These ‘nutrition impact’ symptoms, and the resulting malnutrition, reduce patients’ quality of life (QoL) and length of survival, for example through chemotherapy dose reductions as a result of intolerable chemotherapy side effects [4–6]. Treatment of symptoms contributing to malnutrition in pancreatic cancer is therefore key to improvement in QoL and survival. Our team have previously conducted a two-arm randomised controlled trial investigating the impact of intensive dietary counselling on QoL in upper gastrointestinal (UGI) cancers [7]. In this study, all participants with pancreatic cancer (*n* = 44) experienced at least one nutrition impact symptom prior to commencement of treatment; three-quarters (*n* = 33) of participants experienced more than five symptoms, around half (*n* = 23) experienced more than ten, and one participant experienced 20 different nutrition impact symptoms prior to treatment [8]. Many tumour- and treatment-associated nutrition impact symptoms are not sufficiently controlled with appropriate prescription of medication with chemotherapy-induced nausea and vomiting underestimated (and therefore undertreated) by clinicians [9, 10]. Patients with pancreatic cancer also often experience digestive symptoms associated with pancreatic exocrine insufficiency (PEI) [2]. PEI severity is an independent prognostic determinant, and adequate treatment using pancreatic enzyme replacement therapy (PERT) has been shown to improve survival rates and QoL through alleviation of gastrointestinal symptoms [11, 12]. However, in Australia, PERT is poorly prescribed for patients with pancreatic cancer, with reports of only 21 to 52% of patients with pancreatic cancer receiving it, in part due to a lack of standardised optimal screening pathway to identify PEI [7, 13, 14].

Dietitians are experts in clinical nutrition and are trained to provide nutrition support, advocate and strategise for effective control of symptoms that arise from chemotherapy and PEI [15]. Strategies such as dietary education, counselling and the provision of nutrition support either via the oral route, including the use of oral nutrition supplement drinks or via the enteral route using a feeding tube, are used by dietitians to optimise nutritional intake of patients. Dietitians are important members of the cancer multidisciplinary team; however, evidence suggests that only up to half of patients with pancreatic cancer are consulting with dietitians [16, 17]. Our previous study demonstrated that control group participants with pancreatic cancer who received ‘usual nutrition care’ waited an average of 82 days before first contact with a dietitian, with some never seeing a dietitian [7]. Routine malnutrition screening does occur in most health services across Australia, but barriers to intervention include screening not occurring early enough in a patient’s cancer journey, or the out-of-pocket costs associated with some dietitian services, or the specialist expertise of the dietitian [18–20] An additional barrier to early nutrition intervention is the ability of patients to physically access dietetic services face-to-face [21, 22]. Patient perspectives on early telehealth-based interventions to overcome this barrier were investigated in a qualitative study exploring acceptability of different models of nutrition care delivery to patients undergoing treatment for UGI cancers [21]; given the significant physical and emotional burden that cancer diagnosis and treatment places on patients, the convenience and flexibility of telehealth intervention at home was widely preferred [21, 23].

**Fig. 1:**
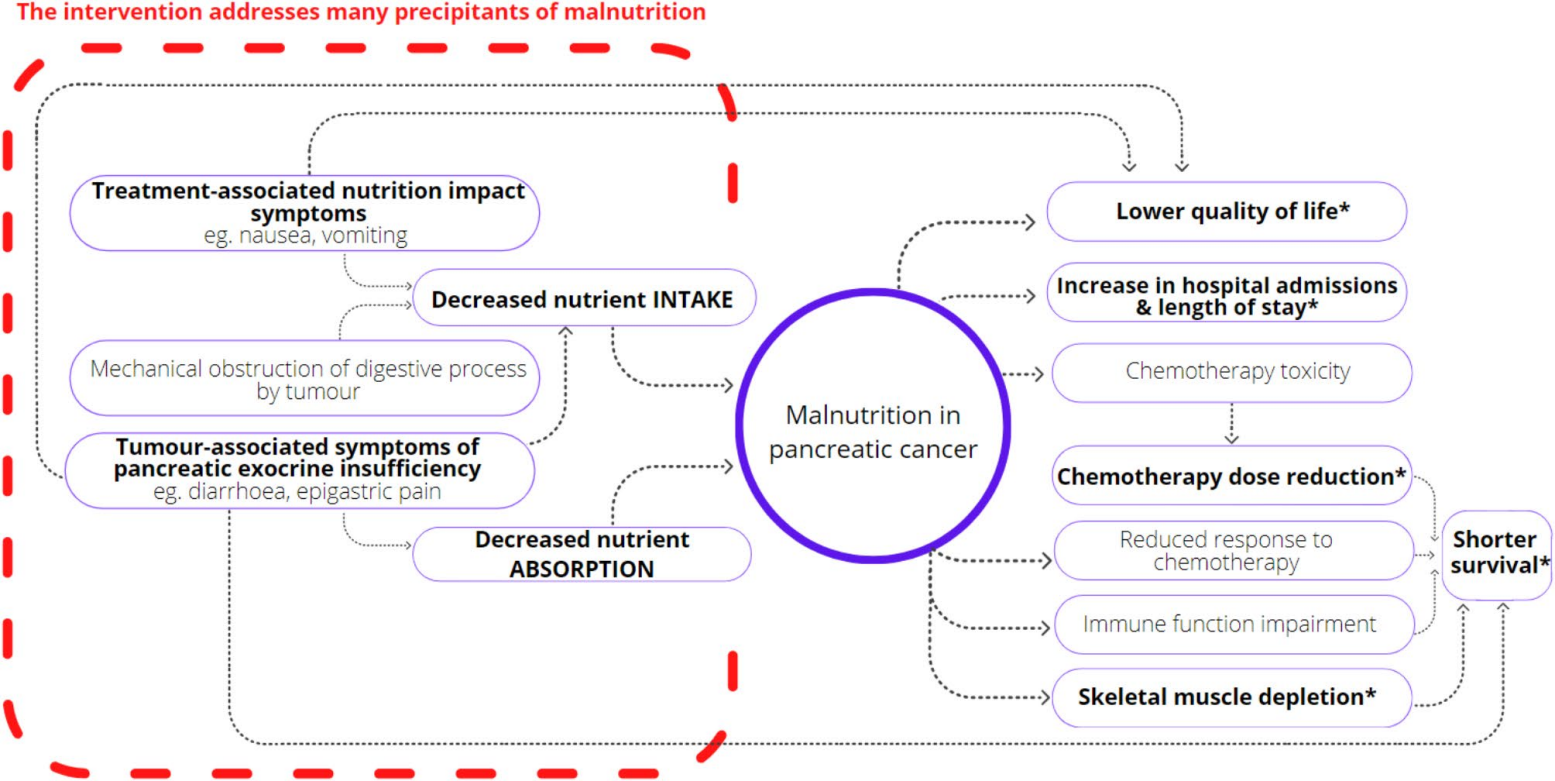
Precipitants and consequences of malnutrition

The aim of this study is to determine if early, intensive nutrition intervention comprising dietary education and counselling, provision of oral nutrition supplement drinks, proactive symptom control and the use of jejunal enteral feeding as needed, can improve health-related quality of life in people with newly diagnosed pancreatic cancer. In addition, compared to usual care, the intervention may improve survival length, chemotherapy dosing, nutrition status, symptom burden and economic measures (Fig. 1).

The proposed intervention involves dietary education and counselling commencing earlier and occurring more frequently than usual care, as well as provision of oral nutrition supplement drinks, effective symptom management and escalation to enteral feeding as needed. It is hypothesised that the intervention will address the many precipitants of cancer-associated malnutrition (denoted in bold text on the left of the circle) and reduce the subsequent risk of poor outcomes associated with malnutrition (denoted in bold on the right of the circle). Outcomes denoted with an asterisk will be measured in this study.

## Methods

### Study design

**Fig. 2:**
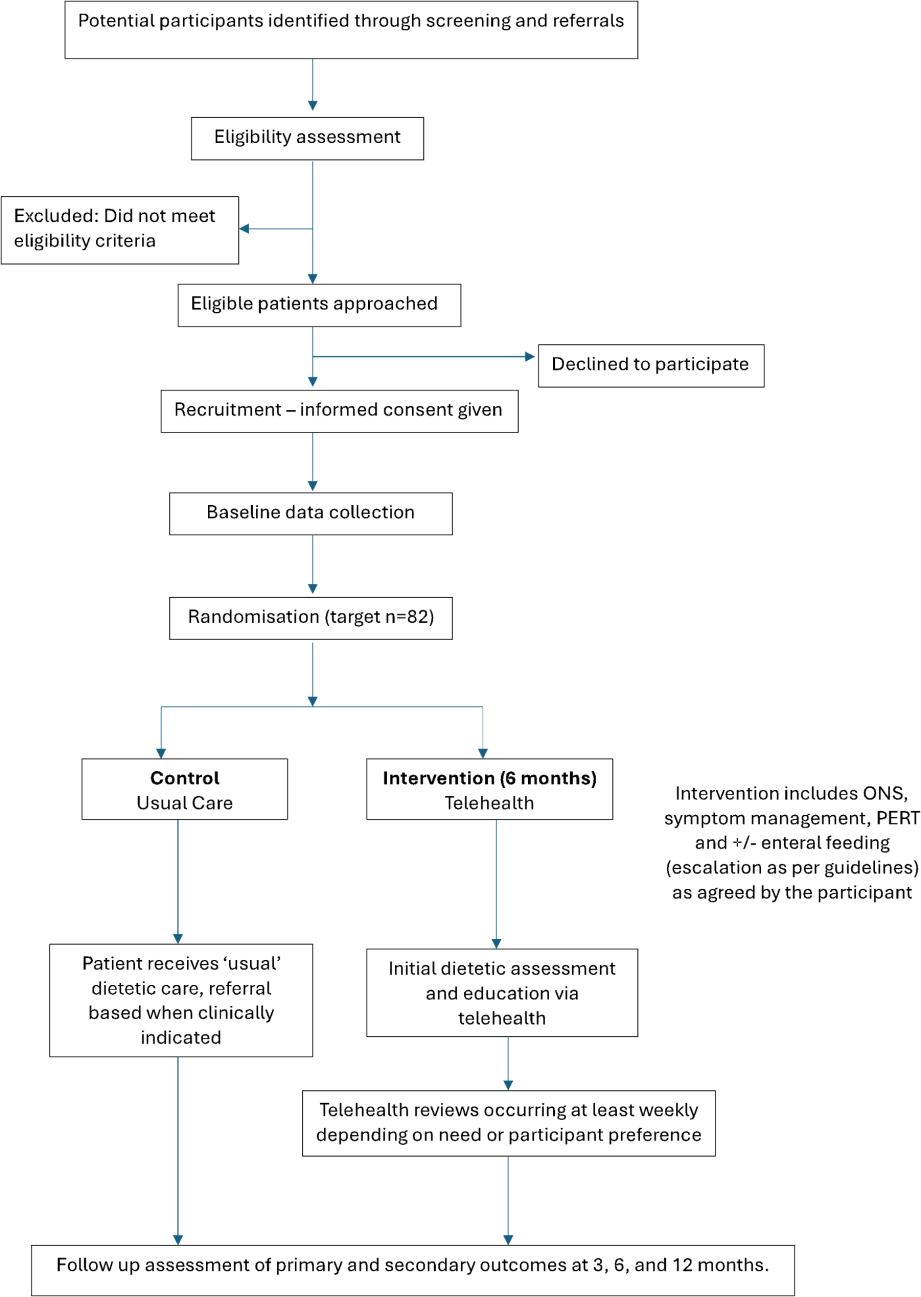
Study flow chart

This study is a two-arm, multicentred, randomised controlled trial. Outcomes will be measured at baseline, three, and six-month follow-up time points. Figure 2 represents the study design and participant flow.

The study flow chart depicts the two pathways; the intervention and usual care group. The usual care group will be screened for malnutrition at their healthcare services and will be referred to a dietitian when clinically indicated. Those in the intervention group will receive weekly telehealth dietitian interventions.

### Participants and setting

This study will recruit patients with newly diagnosed borderline resectable, locally advanced or metastatic pancreatic cancer who will be receiving or have already received one cycle of chemotherapy treatment across Victorian health care services. Regional and rural health care services are included in participating sites to help reduce inequities across cancer care as demonstrated in the Australian Cancer Plan [24].

### Eligibility

Individuals aged > 18 years with a diagnosis of pancreatic cancer by radiological imaging, and/or biopsy and/or multidisciplinary agreement, that are expected to survive at least six months from diagnosis will be screened for eligibility to participate. Patients will also require an Eastern Cooperative Oncology Group (ECOG) Performance Status Scale is two or less to be eligible [25]. Those with neuroendocrine pancreatic cancers or who have previously undergone pancreatic cancer surgical resection will be ineligible. Those who decline or who are unsuitable for systemic chemotherapy, will also be ineligible. Patients will not be eligible to participate if they have serious medical or psychiatric conditions that might compromise protocol-based management as judged by the patient’s treating healthcare team. Individuals with insufficient cognition or knowledge of the English language as determined by the treating healthcare team, who do not have a family member who can assist with English translation to facilitate the completion of outcome data collection or interviews with the study dietitian will also be ineligible to participate.

### Recruitment

Potential eligible individuals will be identified by site study representatives at each health service through their multidisciplinary meetings and oncology or endoscopy clinic lists. Once identified, site study representatives will screen the patient based on the inclusion criteria, and if eligible will contact the patient via phone or in person to invite them to participate. The details of the study will be provided to patients through the Participant Information and Consent Form (PICF). Patients will be invited to discuss their participation in the study with a support person who can assist in the decision-making, such as a family member, friend, or their oncologist or general practitioner. Patients will have the opportunity to ask the investigators further questions about the study as required. To enrol in the study, patients will sign an e-consent form via REDCap or a paper copy of the consent form, which will be documented according to Good Clinical Practice Guidelines [26, 27]. Once enrolment is confirmed, participants will be sent a link to a baseline questionnaire on REDCap or via email with a document attached, with the additional option of completing this over the phone or in person with a research assistant if preferred.

### Randomisation and blinding

The random allocation sequence will be developed by a statistician who has no role in the recruitment of participants or data collection. Dynamic randomisation methods will be employed using STATA 18, based upon dichotomisation of malnutrition risk score at baseline assessment (Patient Generated Subjective Global Assessment Short Form (PG-SGA_SF_) score of < 12 vs. ≥ 12 based on distribution of baseline scores from our previous research) cancer stage (borderline resectable or locally advanced vs. metastatic), and commencement of chemotherapy (yes vs. no) [7, 28, 29]. The randomisation sequence will be generated by the study project managers and kept in a password-encrypted file. The project managers will contact the intervention dietitian and provide the sequential participant registration number. Participants will be informed of their group allocation via phone by the intervention dietitian.

### ‘Usual care’ control group

Routine dietetic care will be received by those participants randomised to the control group, also known as usual nutrition care. Usual care most commonly involves nurse-led malnutrition screening at each chemotherapy visit or acute inpatient admission, with patients at risk of malnutrition referred to a dietitian for nutrition assessment and intervention to optimise nutrition status. The timing and delivery of usual care will differ between health services.

### Intervention group

The intervention will be provided in addition to usual care for a duration of six months. The intervention differs to usual care in terms of the mode, frequency and intensity of dietetic involvement. Dietitians will complete training in Behaviour Change Technique Taxonomy v1 (BCTT – V1) and all aspects of the intervention including symptom management, nutrition counselling, provision of oral nutrition supplement drinks, enteral nutrition regimen development and feeding tube management [30]. To reduce barriers to nutrition care, the nutrition intervention will be delivered via telehealth making it more widely accessible for a larger population, with the mode (telephone or videolink) determined by participant preference [21, 22]. Dietetic consultations will occur weekly at a minimum, as previous studies have displayed that consistent dietetic interactions can create a sense of support and rapport leading to greater engagement and disclosure of symptoms [21]. The intervention dietitian will contact participants as soon as practicable after randomisation to complete the initial nutrition assessment, and to arrange postage of a resource pack to participants. The pack contains an information booklet outlining key contacts, weight record templates, common side effects of treatment and how to manage them, and nutrition intervention strategies and education. The intervention dietitian will refer to the participant booklet during consultations, as a guide for dietary education and counselling. The booklet al.so outlines enteral feeding information including daily care of feeding tubes and a section to document individualised regimens for those that receive a tube. The participant can choose to record information in the booklet, such as medication changes or timing of symptoms, to report back to the intervention dietitian during weekly consults. Participants will also receive a diary to record PERT dosages, a PERT education booklet, and a pill box to store PERT capsules, as well as samples of oral nutrition supplement drinks to trial. Provision of trial supplement drinks will be tailored as required, depending on participants’ medical history e.g. participants with poorly controlled diabetes mellitus.

The intervention dietitian will assist with optimisation of participants’ oral intake and where relevant, use of a feeding tube for supplementary nutrition, each week for the duration of the six-month study period. Dietitian contact with the participant may occur more than once per week if the participant contacts the dietitian e.g. via phone or email with a query, or if there is an escalation in frequency or severity of nutrition impact symptoms.

#### Nutrition optimisation

Intervention dietitians will follow the Academy of Dietetics Nutrition Care Process of Assessment, Diagnosis, Intervention, and Monitoring/Evaluation (ADIME), as adopted by Dietitians Australia, for standardised dietetic care in all nutrition assessments and reviews [31, 32]. This process involves collection of information regarding anthropometry, biochemistry, clinical data, dietary intake, enteral nutrition (where relevant) and consumption of oral nutrition supplement drinks. Individualised nutrition requirements will be calculated using the European Society for Clinical Nutrition and Metabolism (ESPEN) guidelines for nutrition in cancer patients and adapted as required [33]. Diet histories will be quantified using the Easy Diet Diary App to determine total macronutrient and micronutrient intake [34]. Recommendations for oral nutrition supplement drinks and the need for supplemental enteral feeding will be based on adequacy of participants’ oral diet intake, determined using study developed ready reckoners. Reducing deficits in nutrition intake will be prioritised to achieve weight stability and improve nutrition status during treatment. Intervention strategies and goal setting may include the provision of oral nutrition supplement drinks, management of nutrition impact symptoms, dietary education including the utilisation of written education materials, dietary counselling where nutrition recommendations are based on participant’s reported intake and behavioural strategies from the BCTT – V1 and potential for escalation to enteral nutrition where appropriate [30]. Behaviour change techniques will be used to support goal setting e.g. problem solving and action planning. At each participant review, goal achievement will be assessed; goals can be ceased, modified, or continued in addition to the setting of new goals. Behaviour change techniques as described in the BCTT-V1 that are used in each participant interaction will be recorded [30].

#### Oral nutrition support

The intervention dietitian may recommend oral nutrition supplement drinks to help meet an individual’s requirements. The amount to consume each day will be discussed with the participant, aiming to reduce the energy and protein deficit and meet the individual’s nutritional needs. The oral nutrition supplement drinks available to participants are Vital^®^ 1.5, Ensure^®^ Compact 2.4, Ensure^®^ Plus, Resource^®^ Ultra and Resource^®^ Fruit Beverage which will be provided free of charge to participants. This range includes both fruit-based and milk-based options, a peptide-based formula and up to 17 different flavours, allowing greater choice for participants.

#### Enteral feeding

The intervention dietitian will utilise evidence-based guidelines from the European Society for Medical Oncology (ESMO) and European Society for Clinical Nutrition and Metabolism (ESPEN) to determine when escalation to enteral nutrition is required [35, 36]. The ESMO guideline for UGI cancers include considering enteral nutrition to maintain nutrition status when oral feeding (including the use of oral nutrition supplement drinks) is predicted to remain suboptimal [35]. The ESPEN guidelines recommend commencing enteral nutrition when there is suboptimal oral intake of less than 60% of estimated energy expenditure for greater than ten days, where the goal of EN would be to supplement oral intake to meet nutrition requirements [36]. Participants will be assessed for the need of a feeding tube at each dietitian consult and if deemed appropriate by the intervention dietitian, escalation to enteral nutrition will be discussed with the participant. Some participants may require a tube insertion at the commencement of the intervention due to previous substantial weight loss or deteriorating nutrition status. Participants are educated on their first assessment that they may also request this to occur prophylactically. If the participant agrees to an enteral feeding tube insertion, the participant’s primary health service gastroenterology team will be notified by the intervention dietitian to facilitate insertion. If a patient has a borderline resectable tumour, the treating surgeon will be consulted to ensure that tube insertion doesn’t interfere with the participant’s planned surgery. Participants will be supplied with the enteral formula; Abbott Vital^®^ 1.5 cal/mL, a peptide-based, partially hydrolysed, nutritionally complete formula [37]. Alternative formulations may be required as per the dietitian’s recommendation in consultation with the patient. As soon as practical after the feeding tube has been inserted a Home Care Nurse (healthcare company representative) will visit the participant (and/or their family and carers) at home or at their healthcare institution to provide training and education on using and caring for the feeding tube.

The enteral feeding regimen will be established in collaboration with the participant, to meet nutritional demands and to minimise interruptions to the participant’s lifestyle. Regimens will be adapted throughout the intervention phase, depending on changes in nutritional deficits, nutrition impact symptoms arising, weight loss and/or treatment stage. Enteral feeding can be delivered as a continuous or intermittent regime via a feeding pump, or bolus feeds, depending on participant preference and tolerance.

Feeding tubes will be removed at the end of the six-month intervention, unless it remains clinically indicated and a conversation regarding the continuation of feeding has occurred between the participant and their health care team. For participants who choose to continue enteral feeding post conclusion of intervention period, a handover to the nutrition team of the participant’s health service will be provided as they will be responsible for managing the ongoing nutrition care of patients who receive enteral nutrition at home.

#### Nutrition impact symptoms

All nutrition impact symptoms will be recorded for all intervention participants at each consult, and strategies to assist with the management of these will occur through goal setting, delivery of nutrition education and counselling, and recommended prescribed medications.

#### Pharmacological support

To help manage nutrition impact symptoms in a timely manner, all participants will be provided with a standard set of prescriptions upon commencing the study which will be sourced by the site liaison, including prescriptions for PERT (25,000IU microsphere capsules), metoclopramide (10 mg tablets), and ondansetron (8 mg tablets or wafers). The intervention dietitian will be able to guide participants on correct usage as per medical instructions of the prescribed medications to assist in preventing or relieving symptoms. Severity of PEI and the requirement for PERT will be assessed using the American Gastroenterological Association Guidelines, which involves screening for presence of symptoms such as bloating, gas, altered bowel motions, weight loss and steatorrhea [38]. Timing, dosage and correct administration of PERT will be guided by the dietitian in line with oral intake (including oral nutrition supplement drinks), symptoms and/or supplemental enteral feeding and will be re-assessed at each telehealth consultation with dosages/timing adjusted as required. PERT will be administered either orally or with enteral feeds using either a naso-jejunal tube (NJT), percutaneous endoscopic gastro-jejunal tube (PEG-J) or radiologically inserted gastro-jejunal tube (RIG-J). If required, the intervention dietitian may recommend that participants purchase over the counter medications such as aperients or anti-diarrhoeals. All other medications will need to be prescribed by the participants’ treating medical team or general practitioner e.g. opioid analgesics or antidepressants, and will be charted accordingly. The intervention dietitian will work closely with the participant’s treating medical team to escalate the need for further medications as required.

#### Psychological support

The intervention dietitian will complete a brief National Comprehensive Cancer Network (NCCN) Distress Thermometer score with participants at the first assessment and thereafter where deemed appropriate (i.e. where there appears to be a change in the participant’s mood or affect) [39]. Where required, with participant consent, the dietitian will determine the appropriateness of referrals to health service’s social work or psycho-oncology departments as per usual care. The Cancer Council telephone information and support service, SuicideLine and Lifeline phone numbers will be provided to participants at commencement of the study in the participant information pack. Urgent escalation of required psychological support may occur through contacting the area mental health services.

### Hospital admissions

Participants may be admitted to hospital for medical treatment during the intervention period, and may be seen by a dietitian within the local health service as part of usual care. The intervention dietitian will identify patients who have been admitted via the health services’ electronic medical records (EMR); alternatively, the participant may alert the intervention dietitian via phone or email. To ensure the continuum of care, detailed handovers will be provided to the dietitian at the health service by the intervention dietitian in a standardised ISBAR (identification, situation, background, assessment, and recommendation) format using a study developed proforma [40]. Upon discharge from hospital a handover will be provided back to the intervention dietitian.

### Adherence

If a participant misses a planned nutrition assessment or review, the intervention dietitian will make phone or email contact. The consult may be rescheduled or adapted to another telehealth means as required. Adherence to nutrition recommendations that impact on nutrition impact symptoms will be monitored by the intervention dietitian at each telehealth review.

### Data collection

Outcome data will be collected at baseline, three months and six months, in a manner preferred by the participants: either via phone with the blinded data collector, or self-completed via REDCap or email. Physical assessments will be carried out in person at the participant’s home, or their chemotherapy day unit at a time of their preference, by a blinded study data collector.

### Primary outcome

The primary outcome for this study is health-related QoL as measured using the summary score of the EORTC–QLQ-C30 at each follow-up time point [41]. This is a validated, reliable and widely used measure of health-related quality of life in oncology, palliative and supportive care research, including five functional subscales (physical, role, cognitive, emotional, and social) and with a range of domain-specific subscales available that capture symptoms of pancreatic cancer including pain, fatigue, nausea and vomiting, dyspnoea, insomnia, appetite loss, constipation, and diarrhoea [42]. The summary score is determined using the average of all the QLQ-C30 scale and item scores (as above) however not including global QoL and financial impact [43].

### Secondary outcome measures

QoL using the pancreatic cancer specific supplementary module, the EORTC-QLQ-Pan26, EQ-5D-5 L and 12-month mortality are secondary outcome measures [44, 45]. Presence of malnutrition and severity grading will be measured using the Patient Generated Subjective Global Assessment Short Form (PG-SGA_SF_), Global Leadership Initiative on Malnutrition (GLIM) Criteria, and ICD-10 [29, 42, 46]. These tools are commonly used globally to measure the nutrition status of oncology patients [29]. Weight will be assessed to help determine the impact of the intervention on the incidence/degree of weight loss, which is a negative indicator for treatment outcomes and survival [47]. Skeletal muscle mass will be assessed using the gold standard method of analysis of computed tomography (CT) imaging undertaken during routine clinical care at baseline and six months, and using muscle ultrasound and calf circumference at baseline, three months and six months. Low skeletal muscle mass is an independent prognostic indicator and is associated with dose-limiting chemotherapy toxicity [48, 49]. Skeletal muscle function will be assessed using measuring hand grip strength at baseline, three months and six months [50]. The Demoralization Scale–II, a well validated 16-item self-report measure, will be used to assess demoralization [51]. Chemotherapy dose reductions will be measured using relative dose intensity (RDI) which is the ratio of the delivered dose intensity (dose per unit of body surface area per unit of time) to the planned chemotherapy dose and this will be collected in the weekly dietitian consultations and/or through the EMR. An RDI below 85% is considered a clinically significant reduction from planned chemotherapy [52].

### Intervention fidelity and safety secondary outcomes

Intervention fidelity measures will be undertaken to understand the degree to which the intervention was able to be delivered as planned. These include: provision of oral nutrition supplements and consumption, PERT recommendations, medication recommendations, referrals to support services, feeding tube insertions, and feeding tube complications.

### Economic evaluation

Economic measures will be used to inform an economic evaluation, including both costs and measures of utility necessary to undertake a cost utility analysis. Health utility will be measured using the EQ-5D-5 L, which is a generic measure of health-related quality of life that does not include the cancer symptom-specific domains of the EORTC QLQ-C30 [43, 44]. However, use of a generic health utility scale is necessary for calculating the Quality-Adjusted Life Years (QALYs) lived necessary for conducting an economic evaluation consistent with welfare economics theorem. Other measures include; home care service provision (paid and unpaid) using the iMTA MCQ, direct health costs captured using Medicare Benefits Schedule (MBS) and Pharmaceutical Benefits Scheme (PBS) data, direct costs associated with hospital admissions, days spent in hospital, procedures performed in hospital, subjective reports on additional non-MBS health service use, e.g. private dietitian, and costs associated with feeding tube insertions and complications [53]. An incremental cost-utility analysis will be undertaken from the societal (primary), health service (secondary) and patient (secondary) perspectives. Costs will be valued at a 2024 base-year. QALYs lived will be calculated using an Australian utility value set for the EQ-5D-5 L [54]. Bootstrap resampling will be used to calculate a 95% confidence ellipse and cost-effectiveness acceptability curves.

### Power calculation

A two-group comparison power analysis using the primary outcome, a power of 80%, and two-tailed alpha of 0.05 was undertaken. We considered that the minimum standardised effect that would be needed to justify the cost of the intervention would be a delta of 0.50 (large effect). In absolute terms, this is a change of 0.15 in health utility (scale value of 1 = perfect health, value of zero = death) given a standard deviation in our TEND study data of 0.29 for health utility at baseline [7]. With these inputs and considering inclusion of a baseline measurement and two follow-up measurements (3 months and 6 months) with correlation between them of 0.41 (based on data from our previous randomised controlled trial) in the model, we calculated that we would need *n* = 34 participants per group. We increased this requirement by 20% to *n* = 41 per group to account for potential loss to follow-up or missing data.

### Blinded outcomes

The primary outcome measure (QoL) will be collected and analysed by blinded research staff. The baseline QoL survey will be completed prior to group allocation and then at three- and six-months via REDCap. If the participant choses to do this in person, the survey will be completed by alternating research assistants, to ensure this measure remains blinded. In the event the study group is revealed to the research assistant, the survey will be ceased, and another blinded research team member will complete the survey with the participant.

### Database extraction

The intervention dietitians will have access to each hospital’s EMR to allow for data collection and collaboration with multi-disciplinary members to occur. Data extractions from MBS and PBS databases will be completed at the end of the trial where possible.

### Data analysis

#### Primary and secondary trial outcomes

Analyses will be undertaken on an intention-to-treat basis. The primary outcome (EORTC QLQ-C30 summary score) will be compared between groups using a linear mixed model analysis approach, using values collected at three and six months, and adjusting for baseline values of this variable as well as age, sex, cancer stage, and malnutrition risk score (PG-SGA_SF_ score) [29, 43]. Group allocation will be treated as a fixed effect, while individual participants and assessment time points will be treated as random effects. We will conduct relevant checks of distributional assumptions and model fit. Twelve-month mortality will be compared between groups using Cox proportional hazards regression analysis, adjusting for baseline values of health-related quality of life (EORTC QLQ-C30 summary score), as well as age, sex, cancer stage, and malnutrition risk score (PG-SGA_SF_ score) [29, 43]. Multiple imputation will be used in the event of missing data with checking or relevant assumptions for missing-ness to inform the final imputation approach [55].

#### Mediation analysis

Mediation analysis is increasingly used in randomised controlled trials to confirm the hypothesised working mechanism underlying an intervention. We will use path analysis techniques and follow four recommended steps [56]; (i) descriptive statistics, (ii) test of direct effect of treatment on the mediator (iii) testing the indirect (mediating) pathway, and (iv) testing the indirect (mediating) pathway for potential confounding. Both mechanistic and intervention fidelity and safety secondary outcomes will be included in these mediation analyses.

### Data management

Data will be stored in secure, password protected Australian cloud-based storage repositories (LabArchives and REDCap) [57]. Most participant information will have identifiers removed and be replaced by an individual code. Any necessarily identifiable participant information will be available to unblinded chief investigators only. Data will be embargoed from open sharing until the final publication of the primary outcomes paper.

### Protocol deviations

Deviations from this clinical trial protocol may occur and are permitted with rationale of patient safety and wellbeing or considered to be clinically appropriate. All protocol deviations will be recorded and reported to the Chief Principal Investigator or study committee.

### Adverse event reporting

Any untoward medical occurrence or clinical signs in participants or unintended disease or injury, related to the intervention procedures is considered an adverse event. The procedure for investigators reporting any adverse events involves; reporting the event via the electronic case report form (eCRF) through REDCap as soon as possible but no later than 10 working days for adverse events and 24 h for serious events. The adverse event information will be reviewed and approved by the investigator.

### Data safety and monitoring board (DSMB)

An independent multi-disciplinary and multi-site Data Safety and Monitoring Board (DSMB) will be convened to review accumulating trial data to monitor the safety and progress of the clinical trial. This will include reviewing data on recruitment progress, safety data including any adverse events, and protocol deviations. The DSMB will meet every 6 months for the duration of the trial, and will provide recommendations to the study Governance Committee, as needed.

### Ethics and trial registration

This study has undergone a full ethical review by the Human Research Ethics Committee at Monash Health and was approved on 30th of January 2024. Site-specific authorisation will be obtained from all sites prior to recruitment at each site. This trial was registered prospectively on the Australian New Zealand Clinical Trial Registry on 31st January 2024 (Trial ID: CTRN12624000084583).

### Confidentiality

All participant identifying documents will be disposed of appropriately after each participant interaction.

### Dissemination

The Governance Committee will review the trial findings once the study has ended, to assist with interpretation of study results. Study findings will be published in peer-reviewed journals and will be communicated through oral conference presentations to an audience of multidisciplinary health professionals, to inform researchers, health professionals and policymakers. Media outlets (both local and national) will be contacted once the findings are published, to increase exposure. Authors of international best-practice guidelines for management of people with pancreatic cancer will be contacted and provided with copies of our project report and manuscripts.

## Discussion

Malnutrition is commonly associated with pancreatic cancer [58]. Malnutrition, which involves the loss of skeletal muscle mass and function, reduction in immune function and can contribute to chemotherapy toxicity leads to poor outcomes such as QoL and shorter survival [6, 59]. To prevent malnutrition, nutritional intake must meet an individual’s nutrition requirements; this is challenging for people with pancreatic cancer due to the many tumour- and treatment-related impact on nutrient intake and absorption [60]. This study aims to appropriately manage nutrition impact symptoms and provide intensive nutrition support so that participants meet their nutritional needs, which may improve QoL.

Many studies have reported the detrimental consequences that treatment side effects and nutrition impact symptoms have on nutrition status [61, 62]. However, few studies have investigated the effect of dietitian-led interventions using a multidisciplinary approach to improve symptom management on QoL whilst also aiming to improve nutrition status [63]. Identification and management of nutrition impact symptoms are an important part of the dietetic nutrition care process, as symptoms impact heavily on nutritional intake [64]. This study will determine if aligning an intervention to focus on rapid management of nutrition impact symptoms as early as possible, may help to mitigate barriers to participants adhering to dietetic interventions, and thus improve nutrition status and QoL.

PEI is a common issue that impacts on nutritional intake with poor diagnosis and inadequate prescription of PERT by the medical profession [12]. Patients not prescribed PERT may experience a worsening of symptoms, impacting QoL and nutrition status through significant weight loss and malnutrition [65]. In a pilot study of 44 palliative patients with pancreatic cancer prescribed PERT, there was a significant reduction in diarrhoea, pancreatic and hepatic pain, bloating/gas within 1–3 weeks of its initiation [66]. In a large matched case control study, patients with pancreatic cancer who received PERT had a 262% greater adjusted median survival time (95% CI 2.27–3.02) compared to a pancreatic cancer control group who did not receive PERT (*n* = 807 per group) [11]. Dietitians are well placed to advise on PERT dosage and timing, given their expertise in management of gastrointestinal symptoms and understanding of an individual’s dietary patterns. Close guidance by a dietitian on dosage and timing of PERT in accordance with dietary patterns is recommended as the approach to managing PEI in all patients diagnosed with pancreatic cancer [66].

Despite active attempts to manage symptoms with the aim to improve oral intake, there are times when escalation to enteral nutrition along with symptom management may be needed to improve nutrition status. To our knowledge, there are limited published studies in this patient population exploring the effect of the combination of multiple nutrition interventions [67]. The use of enteral nutrition has been shown to be effective in head and neck cancer where oral nutrition is often impossible or very difficult [68, 69]. For patients with pancreatic cancer, oral nutrition may or may not be impacted by obstruction of the gastrointestinal tract; therefore it can become nearly impossible to meet nutritional needs orally, therefore an enteral feeding approach may be beneficial for some patients [3]. Benefits of a jejunal enteral feeding approach have been demonstrated in a recent feasibility study of 31 patients with inoperable pancreatic cancer who underwent surgical jejunal tube placement for supplementary feeding, resulting in improvement in QoL, gastrointestinal symptoms, lean body mass, and weight stability [67].

Intensive weekly monitoring of nutrition impact symptoms and nutrition status will provide information on the effect that consistent therapy has on outcomes such as QoL, survival and nutrition status. It will provide a comparison to usual dietetic care where nutrition interventions are often less consistent and less timely [7]. The use of telehealth as the mode of intervention delivery will allow more participants to engage regularly without contributing to time and cost burdens associated with travel and creates opportunities for participants living in regional areas to access early nutrition intervention to help improve health outcomes [23]. Understanding the effect of a comprehensive nutrition therapy intervention including effective symptom management, provision of oral nutrition supplement drinks, and supplemental enteral feeding, on QoL and health outcomes such as survival length will be enhanced through this study.

## Data Availability

N/A

## Abbreviations

EORTC QLQ-C30: European organisation for research and treatment of cancer quality of life questionnaire-core 30
ED 5Q 5L: EuroQol group 5D-5L instrument
MST: Malnutrition screening tool
QoL: Quality of life
PG-SGA_SF_: Patient-generated subjective global assessment short form
BMI: Body mass index
ICD-10: International classification of diseases 10th revision
ECOG: Eastern cooperative oncology group
PEI: Pancreatic exocrine insufficiency
PERT: Pancreatic enzyme replacement therapy
EMR: Electronic medical record
QALYs: Quality adjusted life years
MBS: Medicare benefits scheme
PBS: Pharmaceutical benefits scheme
CT: Computed tomography
GLIM: Global leadership initiative on malnutrition
ISBAR: Identification, situation, background, assessment, recommendation
NCCN: National cancer council network
NJT: Naso-jejunal tube
RIG- J: Radiologically inserted gastrojejunostomy tube
PEG-J: Percutaneous gastrojejunostomy tube
ESPEN: European society of parenteral and enteral nutrition
ESMO: European society for medical oncology
BCTT: Behaviour change technique taxonomy
ADIME: Assessment, diagnosis, intervention and monitoring/evaluation
PICF: Patient information & consent form
UGI: Upper gastrointestinal
